# A Highly Sensitive Room Temperature CO_2_ Gas Sensor Based on SnO_2_-rGO Hybrid Composite

**DOI:** 10.3390/ma14030522

**Published:** 2021-01-22

**Authors:** Zhi Yan Lee, Huzein Fahmi bin Hawari, Gunawan Witjaksono bin Djaswadi, Kamarulzaman Kamarudin

**Affiliations:** 1 Department of Electrical and Electronics Engineering, Universiti Teknologi PETRONAS (UTP), Seri Iskandar 32610, Malaysia; huzeinfahmi.hawari@utp.edu.my (H.F.b.H.); gunawan.witjaksono@utp.edu.my (G.W.b.D.); 2 School of Mechatronics Engineering, Universiti Malaysia Perlis (UniMAP), Kangar 01000, Malaysia; kamarulzaman@unimap.edu.my

**Keywords:** SnO_2_-rGO hybrid composite, chemoresistive gas sensor, CO_2_, room temperature operation, detection limit

## Abstract

A tin oxide (SnO_2_) and reduced graphene oxide (rGO) hybrid composite gas sensor for high-performance carbon dioxide (CO_2_) gas detection at room temperature was studied. Since it can be used independently from a heater, it emerges as a promising candidate for reducing the complexity of device circuitry, packaging size, and fabrication cost; furthermore, it favors integration into portable devices with a low energy density battery. In this study, SnO_2_-rGO was prepared via an in-situ chemical reduction route. Dedicated material characterization techniques including field emission scanning electron microscopy (FESEM), high-resolution transmission electron microscopy (HRTEM), energy dispersive X-ray (EDX) spectroscopy, Raman spectroscopy, and X-ray photoelectron spectroscopy (XPS) were conducted. The gas sensor based on the synthesized hybrid composite was successfully tested over a wide range of carbon dioxide concentrations where it exhibited excellent response magnitudes, good linearity, and low detection limit. The synergistic effect can explain the obtained hybrid gas sensor’s prominent sensing properties between SnO_2_ and rGO that provide excellent charge transport capability and an abundance of sensing sites.

## 1. Introduction

CO_2_ is an odorless, tasteless, and colorless gas that is not easily detected by human senses. CO_2_ is also an essential air constitution and is associated with plant survival through photosynthesis. There is a great demand for sensitive CO_2_ sensors in various air quality control applications in the healthcare, space application, biotechnology, food industry, and mining field. In the healthcare industry, for example, sensitive CO_2_ sensors are desired in capnography to detect disease at early stages [1,2]. CO_2_ detection is also crucial in the international space station to ensure astronaut health and safety. A sensitive CO_2_ sensor is used to monitor the CO_2_ concentration in the air of the crew cabin during CO_2_ sequestration processes to ensure that CO_2_ is scrubbed [3]. In the bioprocesses sector, CO_2_ monitoring is important where slight CO_2_ concentration changes can affect microbial cells’ morphology, along with their metabolic products and rates [4]. Therefore, it is imperative to develop an effective and highly sensitive gas detection method for trace amounts of CO_2_, even at low concentrations or at room temperature.

Currently, CO_2_ gas sensors can be classified based on their operation mechanisms, such as resistive [5,6], surface acoustic wave (SAW) [7], optical [8,9], gas chromatography [10], and electrochemical sensor [11]. Resistive-type metal oxides have been widely utilized due to their compact size, long lifetime, low cost, and ease of production [5]. As an n-type semiconductor with a wide bandgap of 3.6 eV, tin(IV) oxide (SnO_2_) is the most recognizable metal oxide due to its potential in a wide variety of applications, including solar cells [12], energy storage [13], and gas sensing [14,15]. For CO_2_ sensing, various preparations of SnO_2_ have been explored by researchers. Wang et al. synthesized a SnO_2_ thick film sensor prepared by a coprecipitation method and showed a sensing response of 1.24 toward 2000 ppm CO_2_ at 240 °C [5]. Xiong et al. prepared undoped and lanthanum oxychloride (LaOCl)-doped SnO_2_ nanofibers through electrospinning techniques, where an operating temperature of 300 °C was used to achieve a sensing response of 1.04 and 3.7 toward 100 ppm CO_2_, respectively [16]. Trung et al. reported a CO_2_ sensor based on LaOCl–SnO_2_ nanowires, which showed a higher response (5.6) than that of bare SnO_2_ nanowires (~1.25) upon exposure to 2000 ppm CO_2_ at 400 °C [17]. According to these findings, typical metal oxide-based gas sensors suffer from detection limits near 100 ppm CO_2_. Furthermore, to operate at high temperatures (240–400 °C), heater circuitry is required, thereby limiting their use in miniaturized circuitry design found in a portable device. Moreover, high operating temperatures can also be dangerous for use in flammable environments such as oil fields. This shortcoming has given rise to a strong dependency on a power source and could deteriorate the lifetime of the gas sensor [18]. Therefore, exploring solutions to have low operating temperatures for SnO_2_-based gas sensors is essential to maintain sensor reliability.

Graphene is a carbon allotrope composed of a single layer of sp^2^ carbon atoms connected in a hexagonal structure. Graphene, which was first discovered by K. Novoselov and K. A. Geim in 2004, has recently gained researchers’ attention because of its extraordinary electrical, thermal, and mechanical characteristics [19]. Reduced graphene oxide (rGO) is a candidate for obtaining room-temperature CO_2_ gas sensing properties close to graphene, such as high electron mobility, high signal-to-noise ratio, and suitability for large-scale synthesis [20,21].

Synergistic hybridization of rGO with metal oxide particles has been an effective way to greatly improve gas detection because it combines both materials’ traits and enhances gas sensing capabilities toward various gases. Many composite modifications have been made, such as the formation of SnO_2_/rGO [22,23,24,25,26,27], zinc oxide/reduced graphene oxide (ZnO/rGO) [28], cobalt oxide/reduced graphene oxide (Co_3_O_4_/rGO) [29] and tin-titanium dioxide/reduced graphene oxide/carbon nanotube (Sn–TiO_2_/rGO/CNT) [30]. These composites exhibit good gas detection, including enhanced sensitivity at room temperature. Great structures have been produced via hydrothermal [23] and sol-gel [31] synthetization methods to fulfill the unique advantages of SnO_2_-rGO toward their specific applications.

To date, attempts made to fabricate CO_2_ gas sensors based on metal oxide/rGO hybrid composite are very limited because, unlike common gases such as ammonia, acetone, and ethanol, CO_2_ is rather inert in nature. Hence, this research attempts to address the above problem by leading us to pursue a strategy to realize a better gas sensor for detecting CO_2_ using the aforementioned metal oxide-rGO composite. Additionally, the reaction process involving CO_2_ can be better understood. Meanwhile, since the publicly available reaction process involving air as carrier gas becomes active sensing sites at the surface of the material, that gives an advantage to sensing performance. Nitrogen is used instead to let the reader understand the underlying direct sensing mechanism of hybrid composite towards CO_2_ [5,32,33].

In the current work, the SnO_2_-decorated rGO hybrid composite was synthesized through a facile reduction method. Since most resistive gas sensors that run on the interdigitated electrode (IDE) involves a simple measuring method and easy device design, the above was chosen as the sensing platform for this study in which a thin sensing film was coated directly on a silicon substrate prepatterned with an IDE. Consequently, the SnO_2_-rGO gas sensor’s response toward various concentrations of CO_2_ operated at room temperature was studied, and the performance was compared with that of a bare rGO gas sensor. A comprehensive study of the CO_2_ gas sensing mechanism was also presented.

## 2. Materials and Methods 

### 2.1. Materials and Reagents 

All reagents, including a commercial 0.4 wt.% liquid graphene oxide (Graphenea, San Sebastian, Spain), tin (II) chloride dihydrate (SnCl_2_·2H_2_O), ethylene glycol, ammonium hydroxide, and ascorbic acid (Sigma Aldrich, St. Louis, MI, USA), were analytical grade and were used without further purification.

### 2.2. Preparation of SnO_2_-rGO Hybrid Composite and rGO

**Figure 1:**
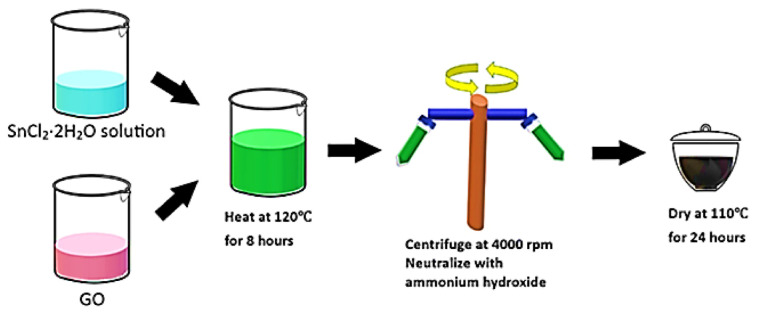
Schematic representation for the preparation of SnO_2_-rGO nanocomposite.

Figure 1 shows the SnO_2_-rGO hybrid composite that was synthesized via a modified reduction method as described in the literature [34]. 10 mL of graphene oxide (GO) were first dispersed in 40 mL of distilled water by ultrasonication for 1 h. After that, approximately 2 g of tin (II) chloride dihydrate (SnCl_2_·2H_2_O) were dissolved in 10 mL ethylene glycol in another beaker under vigorous stirring at room temperature for 1 h, during which the mixture color turned from a transparent to a milky white medium. Subsequently, the precursor solution was added to the GO suspension and heated to 120 °C for 8 h. The as-prepared suspension was then centrifuged and washed with distilled water to remove remaining contaminants such as unwanted ions. A small amount of ammonium hydroxide (10%) was added to neutralize the colloid’s acidity. For comparison, rGO was prepared using the same procedure by replacing tin salt to treat an environmentally friendly yet effective reducing agent, ascorbic acid [35].

### 2.3. Interdigitated Electrode (IDE) Sensor Fabrication

An IDE platform was fabricated through a photolithography process to investigate the sensing performance of the SnO_2_-rGO gas sensor towards CO_2_. Firstly, the silicon wafer was cleaned using a solution containing hydrogen peroxide, hydrogen fluoride, and DI water. A wet oxidation process was then carried out to grow a layer of SiO_2_ on top of silicon wafer before a conductive layer of aluminum is sputtered on top of the substrate. Next, the IDE pattern transfer process was completed from the mask to the top of the silicon substrate. During this process, UV light was passed through a photomask to transfer the IDE pattern on the photoresist covering the top of the silicon substrate. The area which was not covered by photomask was wiped out during the etching process until the IDE structure was formed on the silicon substrate. Then, 2 µL of the as-prepared SnO_2_-rGO in an aqueous solution was deposited on top of the IDE with a sensing area of 5.81 mm^2^ (7 mm width × 0.83 mm height) by using an electronic micropipette (EK80218 PIPETMAN, GILSON, Middleton, WI, USA) through a drop-casting method to produce a thin film. The thickness of the composite film was determined to be approximately ~792.311 nm from the Atomic Force Microscopy observation.

### 2.4. Gas Sensing Measurement

**Figure 2:**
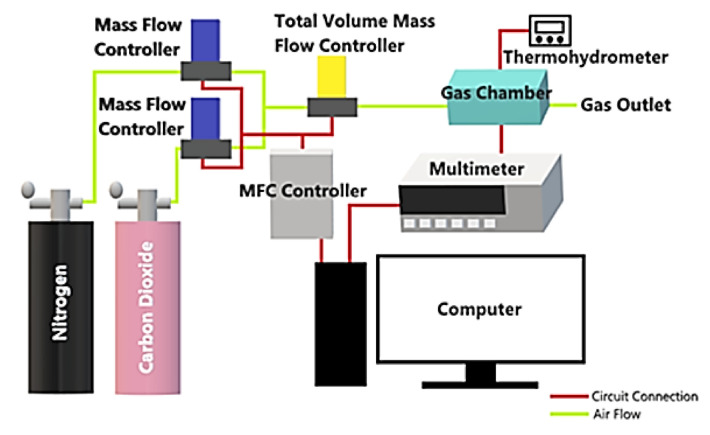
Schematic of the experimental setup for CO_2_ detection.

The testing setup used to evaluate sensor performance toward CO_2_ is schematically illustrated in Figure 2. The data acquisition system consisted of a computer-controlled multimeter (34410A, Keysight, Santa Rosa, CA, USA) connected to the IDE pads through a probe station to measure the gas sensor’s electrical resistance towards CO_2_ and nitrogen exposure at room temperature.

The target gases used in this study were CO_2_ (50 ppm and 500 ppm, Linde, Malaysia) and nitrogen (Linde, Malaysia). Different desired concentrations of gas were purged into an air-tight chamber by diluting the stock CO_2_ gas with known volumes of nitrogen gas using digital mass flow controllers (MFC, DFC26, Aalborg, Orangeburg, SC, USA). The final gas flow was kept at 300 mL/min. Teflon protectors were used in the metal joints of the gas chamber to seal against gas leaks. The room temperature and relative humidity inside the chamber were closely monitored using a digital thermohydrometer. The sensing experiment was conducted in a nitrogen environment and operated under ambient room conditions at 23.5 ± 1 °C and 58 ± 3% RH. Every sensing measurement started with the lowest CO_2_ concentration (5 ppm) and increased gradually until all desired gas concentrations are recorded (5–500 ppm). Before the next target concentration is tested, the gas sensor is purged with nitrogen gas in intervals.

The sensor’s response that reflects the concentration of CO_2_ can be measured by monitoring the resistance across the IDE pads. The gas sensor response is expressed in terms of resistance change upon exposure to gas analytes and nitrogen (Equation (1)):

Response, Rs = (Rg − Ra)/Ra × 100 %(1)

where Ra is the baseline electrical resistance of the gas sensor when exposed to nitrogen and Rg is the resistance in the presence of the target gas. The response time was defined as the sensor’s time to reach 90% of the maximum response change upon exposure to the gas samples. Similarly, recovery time was defined as the time needed to recover back to 10% of the initial baseline value upon exposure to nitrogen gas while cutting off the gas supply from the CO_2_.

### 2.5. Material Characterization

Surface morphologies of the SnO_2_-rGO hybrid composite and rGO sample were examined using variable pressure field emission scanning electron microscopy (FESEM: SUPRA 55VP, Carl Zeiss AG, Oberkochen, Baden-Württemberg, Germany) with energy dispersive spectroscopy (EDX). The morphological analysis was further analyzed by high-resolution transmission electron microscopy (HRTEM: Tecnai G2 F20, FEI, Hillsboro, OR, USA). The Raman spectra of the samples were obtained using a Raman spectrometer (Jobin-Yvon LabRAM, HR800, Horiba, Kyoto, Kansai, Japan, 514 nm). The XPS measurements were recorded on an X-ray Photoelectron Spectrometer (K-Alpha, Thermo Scientific, Waltham, MA, USA, 400 µm) using an Al X-ray radiation excitation source.

## 3. Results

### 3.1. Topographic Details

**Figure 3:**
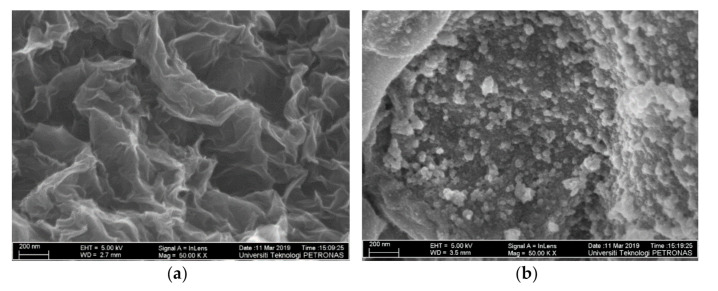
FESEM observations of the (**a**) rGO sheets; (**b**) SnO_2_-rGO hybrid composite.

FESEM was conducted to characterize the surface morphology of the rGO and SnO_2_-rGO composite. As depicted in Figure 3a, under 50 kX magnification, wrinkles made of stacks of single rGO layers can be found everywhere on the surface, which matches with amorphous rGO characteristics [36]. As vividly demonstrated in Figure 3b, synthesized SnO_2_-rGO consists of highly crystalline and spherical constituents, which verify the existence of SnO_2_. With the introduction of an appropriate amount of SnO_2_ cubes into rGO during the facile reduction process, the SnO_2_ species are tightly encased within rGO sheets, and the SnO_2_ small nanocrystals do not agglomerate with each other. SnO_2_ nanocrystals fill in the neighboring rGO spaces, and this wide area of rGO with a crystalline structure of SnO_2_ allows more active sites for gas interaction. 

As an anti-settling agent, ethylene glycol was included during material synthesis to keep rGO stable after losing its hydrophilic characteristics during the GO reduction process [26]. When the solution is coated on IDE, SnO_2_ and rGO fuse together and form a continuous nanocomposite film on the silicon substrate after hardening. The above reduction method produces an organized hybrid structure with distinctive characteristics and paves the way for excellent gas-sensing performance.

### 3.2. Grain Size Analysis

**Figure 4:**
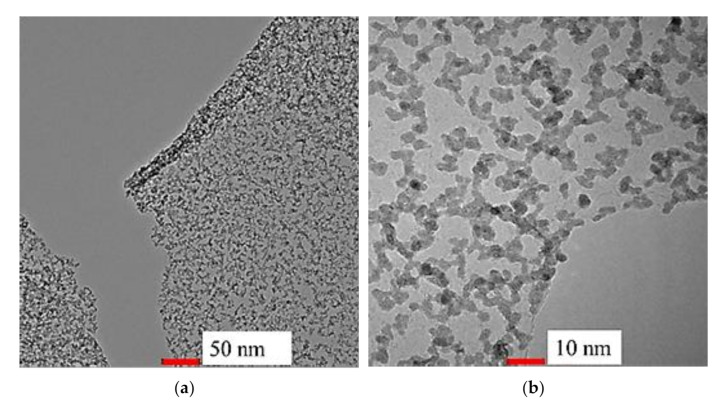

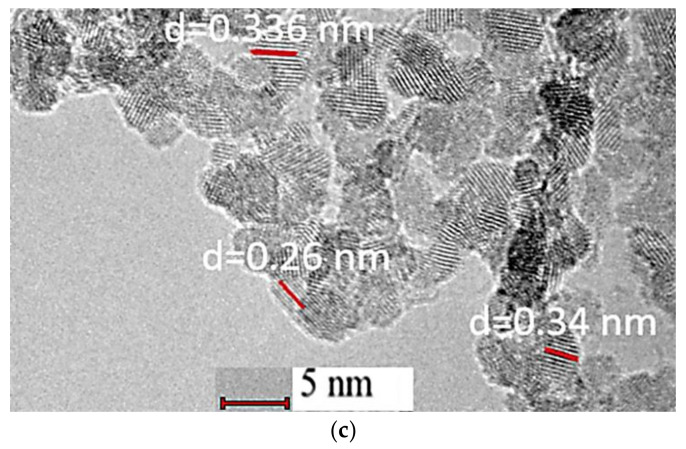
HRTEM image of the (**a**–**c**) SnO_2_-rGO composite with different magnifications to obtain grain sizes of SnO_2_.

In order to study the grain size of SnO_2_-rGO nanostructures, HRTEM analysis was carried out. As shown in Figure 4a,b, the pristine SnO_2_ samples with a diameter ranging from 3–7 nm were observed to be well distributed and properly grown on the transparent rGO sheet. As referred to in Figure 4c, lattice fridges from the sample show two sizes of interplanar spacings consisted of 0.26 and 0.34 nm on average, which corresponds to the (101) and (110) planes from highly crystalline SnO_2_ samples [37]. The morphological characteristics showed above match with the SnO_2_-rGO composite found in previous studies [38,39].

### 3.3. Elemental Composition

**Figure 5:**
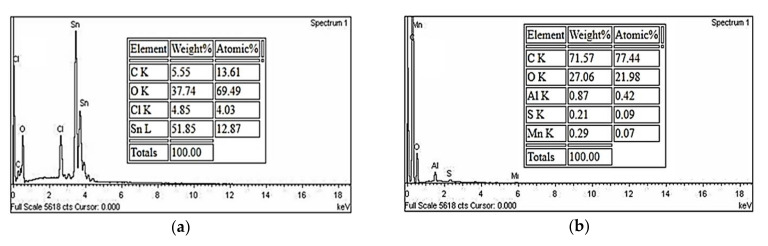
EDX spectra of the fundamental elements found in the (**a**) SnO_2_-rGO composite and (**b**) rGO.

EDX spectrometry was used to identify the elemental composition of the analyzed sample. As shown in Figure 5a, the tin content was 51.85 wt.%, to confirm the existence of SnO_2_ in the hybrid composite film through the reduction route. Moreover, the weight ratio of carbon to oxygen found in SnO_2_-rGO was 0.147, which was less than the 2.645 ratios found in rGO alone, as clearly shown in Figure 5. Content of oxygen element found in hybrid composites is higher because SnO_2_ dopant contains oxygen anions and oxygen residues after reduction of GO within the SnO_2_-rGO composite [40,41,42]. The occurrence of carbon (C), oxygen (O), and tin (Sn) further confirm the formation of SnO_2_-rGO during the in-situ reduction process.

### 3.4. Material Crystallinity, Orientation, and Phases Analysis

**Figure 6:**
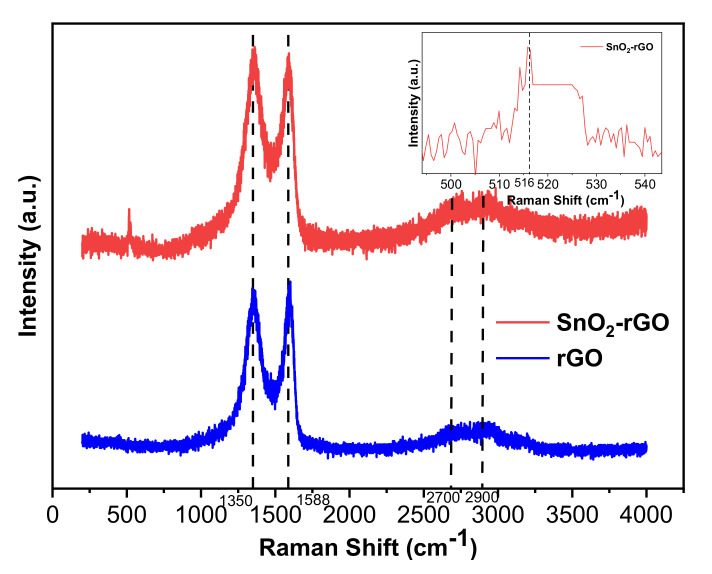
Raman spectra of SnO_2_-rGO and rGO (the inset shows the S1 band that belongs to SnO_2_).

Nondestructive Raman spectra can provide insight into the defects and disorders found in carbon-related structures after a reduction process. As shown in Figure 6, both SnO_2_-rGO and pristine rGO share two prominent bands in common: the D band within the range of 1350 to 1361 cm^−1^ and the G band between 1588 and 1592 cm^−1^, which matches the characteristics of pristine graphene [27,43]. The D peak with high amplitude indicates an A_1g_ breathing mode due to defects and disordered regions induced by functional groups with the sp^2^ hybridized carbon [43]. The G band’s position is shifted to the right, indicating that the better restoration of the carbon plane’s electronic structure is due to the doping effect between SnO_2_ and rGO [27]. The G peak corresponds to an E_2g_ breathing mode from the C-C bond stretching of sp^2^ carbonaceous-based graphene material [38]. Two weak peaks, referred to as second-order D and G (2D and 2G) bands, were also observed overlapping between 2700 and 2900 cm^−1^. In this case, the material has a multilayer structure, which further reinstates that the facile chemical routes successfully synthesize high-density rGO [25,43]. The 2D and 2G peaks will appear regardless of defects in the material.

The intensity ratio (I_d_/I_g_) provides insight into the reduction of GO, which helps determine the structural disorder of the synthesized material. After the addition of SnO_2_ to rGO, the intensity ratio of the D band and G band is 1.006, higher than the 0.993 from rGO. The improved intensity ratio implies that the synergistic effect of the SnO_2_-rGO hybridization creates more disorder in terms of vacancies and grain boundaries and decreases the size of the sp^2^ carbon domain [25,27,44]. GO can be efficiently reduced to a higher degree with tin salt rather than ascorbic acid. The D and G bands’ good intensity ratio and position indicate that SnO_2_-rGO could provide efficient electron transfer at interfaces during the gas sensing process.

In the inset of Figure 6, the wideband peaking at 516 cm^−1^ in the highlighted region relates to S1 of SnO_2_. It exists due to disorder activation and reflects small-sized SnO_2_ crystalline structures within the hybrid composite [25].

### 3.5. Chemical Bonding States

**Figure 7:**
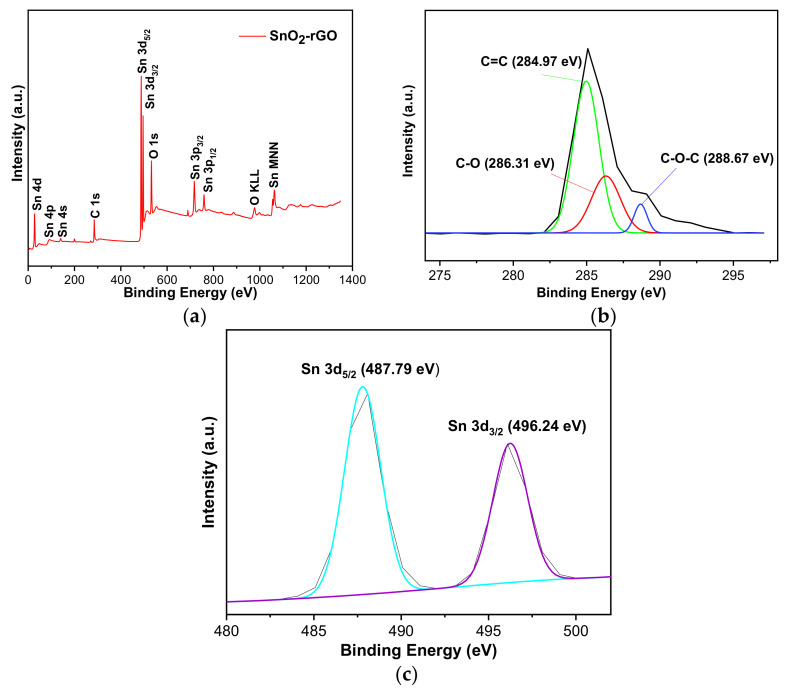
XPS spectra of the SnO_2_-rGO hybrid composite: (**a**) wide survey spectrum, (**b**) C 1s spectra, and (**c**) Sn 3d spectra.

XPS was carried out to determine the chemical states and elemental composition found in SnO_2_-rGO composite. The full XPS survey spectrum in Figure 7a identifies C 1s (285.28 eV), O 1s (532.13 eV), and Sn 3d (487.79 eV) spectra, which confirms the presence of C, O, and Sn elements. Figure 7b shows three peaks of the C 1s spectrum at 284.9 eV, 286.3 eV, and 288.7 eV, which are mainly attributed to C–O, C=C, and C–O–C found in rGO that contains residual oxygen after the reduction process [45]. Moreover, two dominant peaks of the Sn 3d spectrum at 487.79 eV and 496.24 eV were observed, which refer to Sn 3d_5/2_ and Sn 3d_3/2_ in Figure 7c. Three relatively weak peaks of Sn 4d, Sn 3p_3/2,_ and Sn 3p_1/2_ were observed at 28.08 eV, 717.08 eV, and 759.08 eV, respectively. The peak distance between the two Sn 3d spectra was 8.1 eV. The Sn peak location and peak distance confirm the presence of SnO_2_ anchored on the hybrid composite [26]. Additionally, the O 1s spectrum’s binding energies are attributed to carbon atoms with C–C, C-O, and C-OH bonds. These results match the Raman spectra results described earlier, which reinforces that the simultaneous reduction of GO and formation of SnO_2_ nanoparticles were successfully performed using SnCl_2_ molecules to form the hybrid composite.

### 3.6. Gas Sensing Characterization

**Figure 8:**
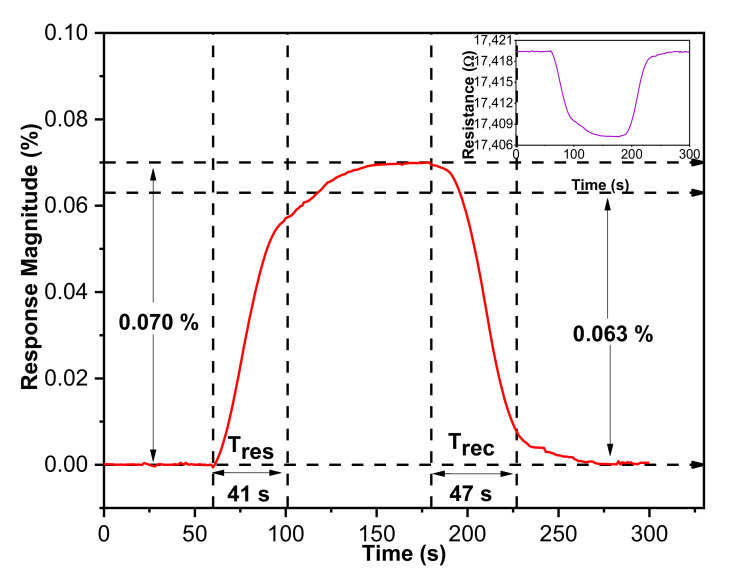
Response transients of the nanocomposite IDE gas sensor toward 5 ppm CO_2_ at ambient room temperature (the inset shows the variation of resistance as a function of time).

The direct-current resistance changes of the IDE-based gas sensor related to CO_2_ gas concentration were monitored to understand sensing kinetics. Figure 8 shows the dynamic response and recovery curve of a SnO_2_-rGO hybrid composite resistive sensor toward 5 ppm CO_2_ gas at room temperature. For 120 s, the hybridized SnO_2_-rGO sensor underwent a negative exponential response curve toward CO_2_. The hybrid structure response was instantaneous for 60 s before transitioning to a steady-state, which indicated saturation of CO_2_ absorption at a response magnitude of 0.070%. According to the response curve, the response and recovery time of SnO_2_-rGO toward 5 ppm CO_2_ were 41 and 47 s, respectively. This gas sensor shows a shorter response/recovery time than those reported using SnO_2_ based sensor, with values of 146 s/50 s and 92 s/98 s, respectively [5,16]. Besides that, this hybrid gas sensor enhanced gas sensitivity towards a low concentration of CO_2_ without dependence on high operating temperature.

The inset in Figure 8 shows the measured sensing curve’s representation in terms of resistance versus time. Sensor resistance decreases once the sensor is exposed to reducing gas CO_2_, which indicates that the hybrid composite shows dominant traits similar to n-type material. When nitrogen gas was alternated with CO_2_ as the main gas source, the hybrid sensor showed good recoverability characteristics where the response managed to recover back to the initial value.

**Figure 9:**
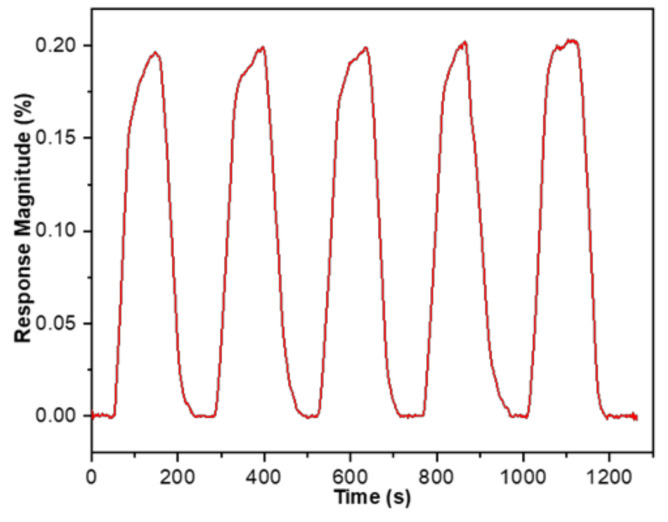
Repetitive response curves of the SnO_2_-rGO composite sensors to 5 continuous cycles of 10 ppm CO_2_ at room temperature.

The repetition test using CO_2_ gas at room temperature was also studied. The SnO_2_-rGO hybrid nanocomposite sensors were exposed to 10 ppm gas on and off for 5 consecutive cycles, and the average response magnitude was 0.200%. As presented in Figure 9, the overall response times of the SnO_2_-rGO nanocomposite upon exposure to CO_2_ with a 10 ppm concentration were kept less than 60 s, and their recovery times upon exposure to nitrogen were also less than 69 s. This behavior is very promising since even for a very low concentration of 10 ppm, the sensor responds consistently in terms of response amplitudes, response times, and recovery times. Simultaneously, the hybrid gas sensor demonstrates that the response can be recovered back to the baseline value for five cyclic periods. This might reflect the fact that CO_2_ can be easily and repeatedly desorbed from the surface of a SnO_2_-decorated rGO gas sensor after the adsorption process. 

**Figure 10:**
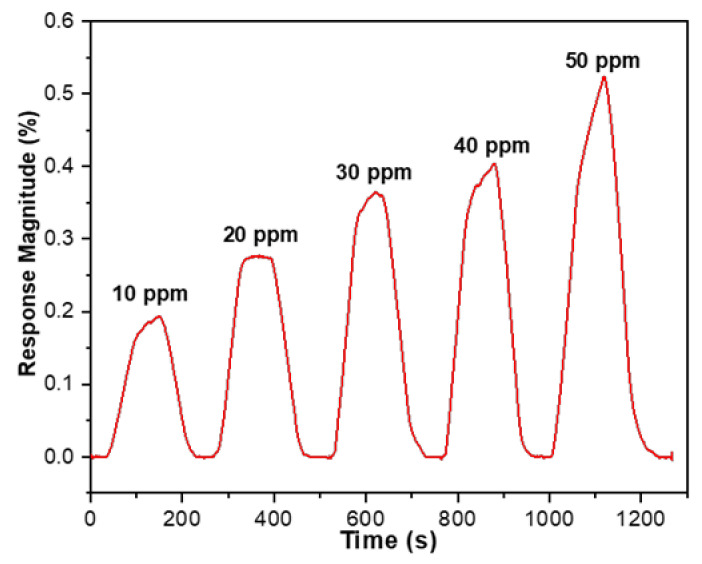
Dynamic responses to transient cycles with the SnO_2_-rGO nanocomposite upon exposure to five different concentrations of CO_2_ gas (10 ppm, 20 ppm, 30 ppm, 40 ppm, 50 ppm) at room temperature.

To further verify the SnO_2_-rGO hybrid nanostructure’s sensitivity towards a wider concentration range, successive responses to transient cycles in relation to low CO_2_ concentrations from 10 ppm to 50 ppm were recorded within 1300 s. According to the Figure 10 curve, the response change of the SnO_2_-rGO sensor is proportional to the concentration of CO_2_, ranging from 10 ppm to 50 ppm. No baseline drift is seen during the gas sensing experiment with the same cyclic period.

**Figure 11:**
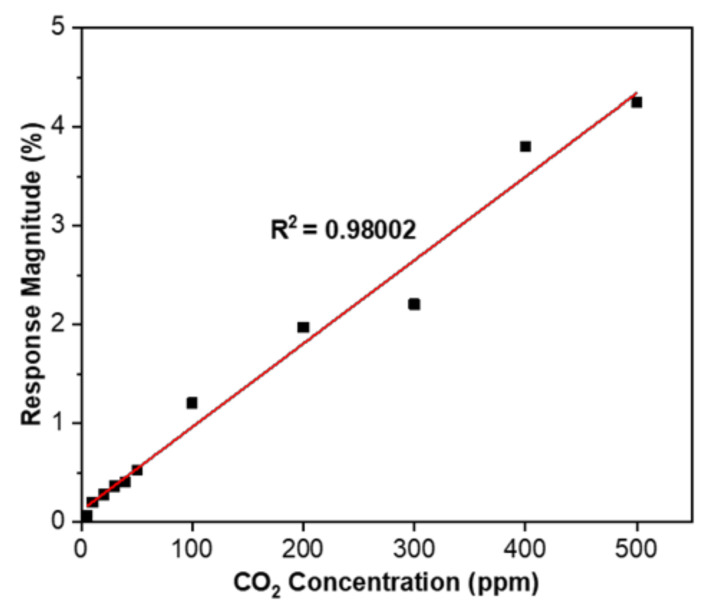
Linear graph showing the relationship between the SnO_2_-rGO sensor response magnitude and CO_2_ concentration.

A SnO_2_-rGO gas sensor’s sensitivity was also investigated upon exposure to 5–500 ppm CO_2_ at room temperature. As shown in Figure 11, the experimental results reveal a good linear relationship between response change and gas concentration. The sensitivity is measured from the slope of the linear graph between the sensor response and gas concentration. From this linear fitted graph, the sensitivity is 0.00845 ppm^−1^ and the linear regression equation is expressed as y = 0.00845x + 0.11832 with R^2^ = 0.98002.

**Figure 12:**
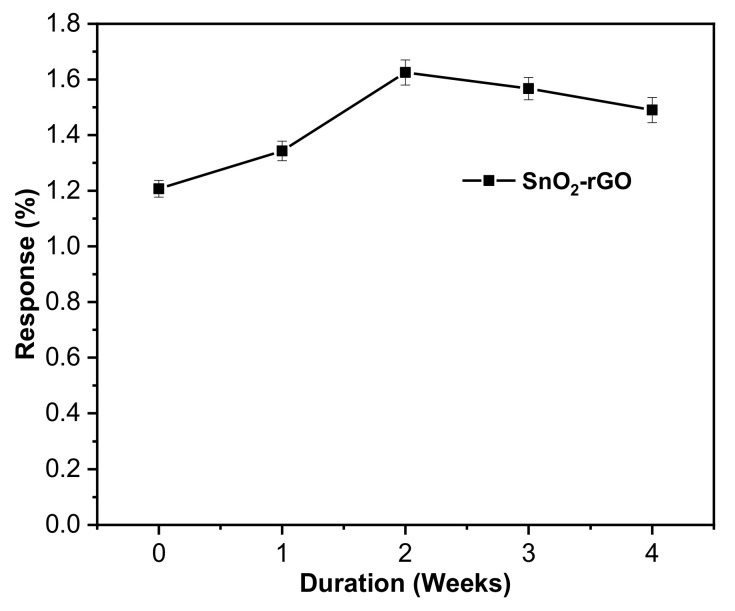
Stability of the sensor response toward 100 ppm CO_2_ over 28 days.

Response stability is a vital statistic for evaluating the functionality of practical gas sensors after multiple uses. To further evaluate the gas sensing performance of SnO_2_-rGO for a month, the as-fabricated sensor is kept under ambient conditions (58 ± 3% RH) at room temperature for testing purposes. As shown in Figure 12, the standard deviation of the CO_2_ gas sensor response change at a testing concentration of 100 ppm every 7 days for 4 weeks is 0.171. The sensor responds consistently to the same concentration of CO_2_ because the sensing material is less affected by chemical change when exposed to the atmosphere for at least a month. Interestingly, as exposure time extended beyond week 2, environmental factors such as dust particles, water vapor, and oxygen molecules tend to occupy active sites and hinder chemisorption, leading to a slight deterioration in response amplitude at a rate of 5.65%/week [27].

**Figure 13:**
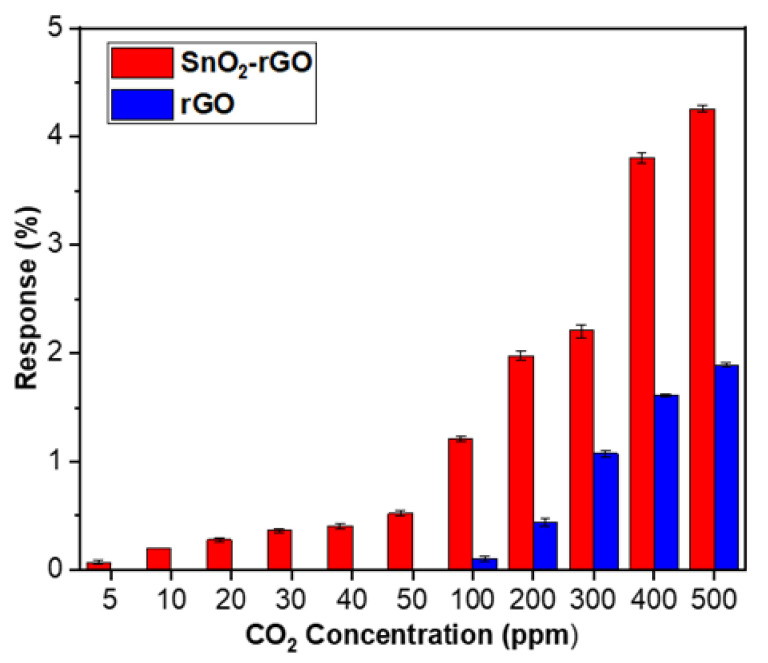
Response magnitudes of the SnO_2_-rGO nanocomposite and rGO gas sensors upon exposure to CO_2_ gas with various concentrations from 5 to 500 ppm at room temperature.

As shown in Figure 13, the SnO_2_-rGO hybrid composite shows good sensitivity to CO_2_ gas even at testing concentrations as low as 5 ppm, whereas rGO shows no sign of response to the same gas analytes a higher concentration of 50 ppm. The response of SnO_2_-rGO increases slowly from 5 ppm to 50 ppm, and then later increases drastically from 100 ppm until it reaches its peak at 500 ppm CO_2_. A comparison of the results indicated that the SnO_2_-rGO hybrid composite gas sensor offered the highest response magnitude and dynamic detection range with the lowest detection limit compared with those of the rGO sensor. This suggested that the catalytic activity of hybridized SnO_2_-rGO preserved more adsorption sites, which improved the sensing performance toward CO_2_.

**Figure 14:**
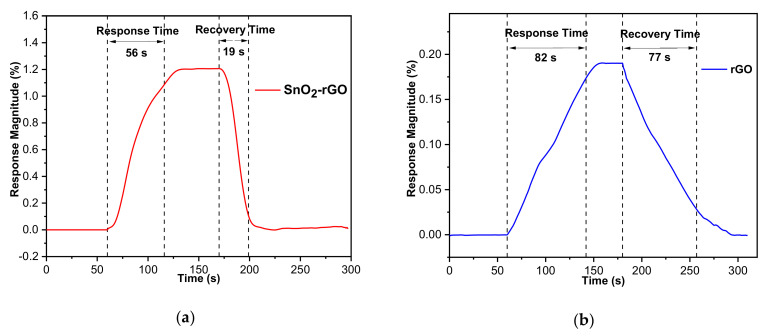
Sensing response towards 100 ppm CO_2_ operated at a relative humidity of 58% RH and at room temperature. Dynamic response curve of (**a**) SnO_2_-rGO and (**b**) rGO gas sensors.

The sensitivity difference of the hybrid composite and rGO sensors is understandable by comparing the dynamic responses to both materials’ transient cycles upon exposure to 100 ppm CO_2_ gas at room temperature. Figure 14 illustrates the sensing response curves for the SnO_2_-rGO and rGO gas sensors exposed to 100 ppm CO_2_ over time. The SnO_2_-rGO hybrid composite gas sensor reads 1.206%, which was 6.7 times better than that of pristine rGO (0.179%). Adding SnO_2_ into rGO provided a high surface area that increased contact with the gas and contributed to enhanced sensing. SnO_2_-rGO achieved a fast response and recovery time of 56 s and 19 s, respectively, toward 100 ppm CO_2_, compared with 82 s and 77 s for an rGO sensor at room temperature. The SnO_2_-rGO hybrid composite gas sensor yields better overall performance because the influence of SnO_2_ in the hybrid composite decreases the conductivity of rGO, forming a comparatively high response change of the sensor [27].

The sensing performance of the fabricated SnO_2_-rGO and rGO gas sensors was compared with resistive-based CO_2_ gas sensors reported in other literature, as shown in Table 1. The nanocomposite sensor detects 5 ppm CO_2_ when operating at room temperature, which is the lowest among previously reported gas sensors, and confirms that the SnO_2_-rGO hybrid nanostructure can ameliorate the detection limit of CO_2_ sensing. In addition, room operating temperature detection is achieved through linkage of SnO_2_ and rGO, as compared to SnO_2_ counterparts.

### 3.7. Room Temperature Sensing Mechanism of the SnO_2_-rGO Gas Sensor

The gas sensing curve based on resistance changes when exposed to the test gas was broken down into three phases to obtain better insight into the above gas sensing mechanism: stabilization, adsorption/response, and recovery. The surface condition plays a key role in gas sensing properties. As the gas sensor is continuously exposed to nitrogen gas for 60 s, the surrounding gas mixture, especially oxygen molecules, is excluded from forming oxygen species such as $O_{2}^{-},{\;O}^{-}$ and $O^{2-}$ in the near-surface region [5,23]. When there is no reacting gas, lesser electrons get enough energy to jump into the conduction band and travel across the junction, while holes are left behind. The accumulation of charge at the interface slowly builds up intermediate space charge layers. The hybrid composite gas sensor will not experience further charge in resistivity when the surface is saturated with nitrogen. 

**Figure 15:**
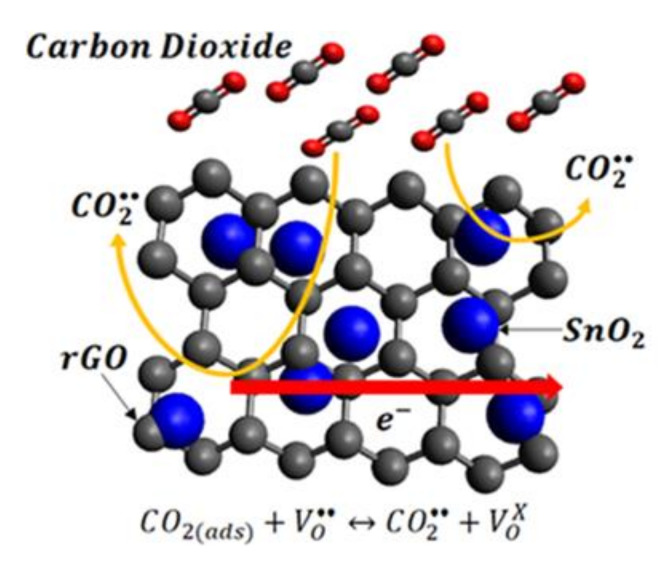
Schematic illustration of the proposed sensing mechanism of SnO_2_-rGO hybrid during CO_2_ detection.

The interaction between sensing film and CO_2_ in the absence of air is illustrated in Figure 15. During the response stage, CO_2_ as a weak reducing gas is partially ionized while reacting with oxygen vacancy sites, $V_{0}^{••}$. The gain of electrons from CO_2_ creates a change of carrier concentration, thereby decreasing its overall resistance. The decline of the sensor’s electrical resistance upon CO_2_ introduction is in agreement with the results observed in CO_2_ detection using SnO_2_ in an oxygen-deficient background [5,16]. At this stage, the electron transfer rate on the sensing film’s surface due to gas adsorption reflects the concentration of gas molecules. Once the gas sensor plateaus signal out, it clearly indicates that the gas sensor is saturated with the target gas. At this moment, nitrogen gas will be purged into the chamber and replaces the CO_2_ molecules on the sensing surface, which results in the desorption of CO_2_ and is responsible for the change of the resistivity back to the baseline. The sensing response upon exposure to CO_2_ is visible but becomes difficult to observe as fewer CO_2_ can undergo ionosorption without oxygen molecules’ influence.

rGO is a highly conductive material, which is attributed to its honeycomb-like arrangement of carbon atoms. This structure allows electrons to travel in a long mean free path while being less likely to be interrupted, thus reducing the gas sensor’s resistance. However, the interaction between rGO and CO_2_ is weak [32] due to the wrinkles restricting the gas sensor potential from having a sensitivity down to a single atom. On the other hand, SnO_2_ and rGO hybrid composites have different but complementary gas sensing characteristics, where SnO_2_ acts as a spacer to avoid the stacking of rGO sheets and rGO overcomes the agglomeration tendency of SnO_2_, which is reflected by the small crystalline structure with diameters of 3 to 7 nm on the surface of the hybrid composite [27]. The functionalization of rGO with the addition of SnO_2_ creates a 3D hierarchical nanostructure with a high surface-to-volume ratio where more surface atoms can interact with the target gas and provides a large, accessible surface area for gas adsorption [23]. 

The facile synthesis process of the hybrid composite by homogeneously blending SnO_2_ into rGO encourages better electron transport and gas adsorption. rGO and SnO_2_ exhibit p-type and n-type semiconducting characteristics, respectively, due to their hybridization effect [24]. Since the hybrid composite is composed of SnO_2_ and rGO with different band gaps, band bending is formed at p-n heterojunctions with a charge gradient that obstructs electron transfer; the above causes a significant change in electrical properties before and after exposure to the target gas at room temperature when electrons, as the majority carriers, are induced or attracted away from the hybrid structures. Environmental factors such as oxygen content and humidity, along with selectivity of SnO_2_/rGO will be studied in future research. 

According to the analyses above, we can conclude that the dominance of the overall room temperature gas sensing performance of the hybrid composite toward CO_2_ molecules with good response and recovery properties is attributed to the high conductivity, an abundance of active sites, and p-n heterojunctions.

## 4. Conclusions

In summary, a facile synthesis route for a hybrid SnO_2_-rGO nanocomposite was conducted, and the material was placed on an IDE platform as a novel room temperature CO_2_ gas sensor device. Due to the synergistic effect from blending the near metallic conductivity of rGO and the metal oxide, the gas sensor showed improvement in a sub-ppm-level detection limit (5 ppm) and excellent CO_2_ sensing at room temperature and 58% RH. The linear relationship between the sensor response and CO_2_ concentration was R^2^ = 0.98002 with a slope of 0.00845 ppm^−1^. The relative response change of the hybrid composite sensor toward 100 ppm CO_2_ was 1.206%, which is 6.7 times better than that of pristine rGO (0.179%). The excellent gas interaction of the SnO_2_-rGO hybrid composite could be attributed to its high conductivity, a wide area of active sites, and p-n heterojunctions. In addition to such a synergistic hybridization of two promising sensing materials (SnO_2_ and rGO), its further advantages, such as a facile fabrication, low production cost, and low power consumption, the gas sensor has the potential for the development of portable CO_2_ gas sensor working at room temperature.

## Figures and Tables

**Table 1:** Overview of the sensing performance of CO_2_ gas sensors.

Sensing Material	Synthesis Method	Tested CO_2_ Concentration @ Operating Temperature	Response	Reference
Graphene sheet	Mechanical cleavage	100 ppm @RT	25%	[32]
Graphene/Y_2_O_3_ quantum dots composite	Electrochemical exfoliation of graphite	35 ppm @RT	1.08	[33]
La_1−x_Sr_x_FeO_3_	Sol–gel method followed by annealing	2000 ppm @380 °C	1.25	[46]
SnO_2_	Coprecipitation method	2000 ppm @240 °C	1.24	[5]
LaOCl-SnO_2_ nanofibers	Electrospinning technique	1000 ppm @300 °C	3.7	[16]
SnO_2_–LaOCl nanowires	Surface coating technique	2000 ppm @400 °C	5.6	[17]
CuO/ZnO	Hydrothermal method	10,000 ppm @320 °C	0.44	[47]
SnO_2_-rGO (current work)		5 ppm @RT	0.07%	
rGO (current work)		100 ppm	0.179%	

## Data Availability

Data is contained within the article. The data presented in this study are available on request from the corresponding author after obtaining permission of authorized person. The data are not publicly available due to data confidentiality resulting from the requirements of the accreditation.
